# Biochemical Screening of Five Protein Kinases from *Plasmodium falciparum* against 14,000 Cell-Active Compounds

**DOI:** 10.1371/journal.pone.0149996

**Published:** 2016-03-02

**Authors:** Gregory J. Crowther, Heidi K. Hillesland, Katelyn R. Keyloun, Molly C. Reid, Maria Jose Lafuente-Monasterio, Sonja Ghidelli-Disse, Stephen E. Leonard, Panqing He, Jackson C. Jones, Mallory M. Krahn, Jack S. Mo, Kartheek S. Dasari, Anna M. W. Fox, Markus Boesche, Majida El Bakkouri, Kasey L. Rivas, Didier Leroy, Raymond Hui, Gerard Drewes, Dustin J. Maly, Wesley C. Van Voorhis, Kayode K. Ojo

**Affiliations:** 1 Division of Allergy & Infectious Diseases, Department of Medicine, University of Washington, Seattle, Washington, United States of America; 2 Tres Cantos Medicines Development Campus, GlaxoSmithKline, Tres Cantos, Madrid, Spain; 3 Cellzome GmbH, Molecular Discovery Research, GlaxoSmithKline R&D, Heidelberg, Germany; 4 Department of Chemistry, University of Washington, Seattle, Washington, United States of America; 5 Structural Genomics Consortium, University of Toronto, Toronto, Ontario, Canada; 6 Drug Discovery, Medicines for Malaria Venture, Geneva, Switzerland; Université Pierre et Marie Curie, FRANCE

## Abstract

In 2010 the identities of thousands of anti-*Plasmodium* compounds were released publicly to facilitate malaria drug development. Understanding these compounds’ mechanisms of action—i.e., the specific molecular targets by which they kill the parasite—would further facilitate the drug development process. Given that kinases are promising anti-malaria targets, we screened ~14,000 cell-active compounds for activity against five different protein kinases. Collections of cell-active compounds from GlaxoSmithKline (the ~13,000-compound Tres Cantos Antimalarial Set, or TCAMS), St. Jude Children’s Research Hospital (260 compounds), and the Medicines for Malaria Venture (the 400-compound Malaria Box) were screened in biochemical assays of *Plasmodium falciparum* calcium-dependent protein kinases 1 and 4 (CDPK1 and CDPK4), mitogen-associated protein kinase 2 (MAPK2/MAP2), protein kinase 6 (PK6), and protein kinase 7 (PK7). Novel potent inhibitors (IC_50_ < 1 μM) were discovered for three of the kinases: CDPK1, CDPK4, and PK6. The PK6 inhibitors are the most potent yet discovered for this enzyme and deserve further scrutiny. Additionally, kinome-wide competition assays revealed a compound that inhibits CDPK4 with few effects on ~150 human kinases, and several related compounds that inhibit CDPK1 and CDPK4 yet have limited cytotoxicity to human (HepG2) cells. Our data suggest that inhibiting multiple *Plasmodium* kinase targets without harming human cells is challenging but feasible.

## Introduction

While screens of compound libraries for anti-*Plasmodium* activity are nothing new [1], there has been a recent trend toward public disclosure of all hit compounds arising from these screens [2–4]. These disclosures facilitate follow-up studies of these “cell-active” compounds and accelerate progress toward new antimalarial drugs. Nevertheless, many challenges remain in developing compounds with activity against culture-grown *Plasmodium* cells into clinically effective drugs [5]. Among these is identifying the compounds’ mechanism of action, i.e., the specific molecular targets by which they kill the parasite. While knowledge of compounds’ targets is not absolutely necessary for drug development, it can enable detailed protein structure studies, inform work on toxicology and acquisition of resistance, and hasten identification of suitable “backup compounds” [5].

*Plasmodium* kinases have great potential as drug targets. Despite the ubiquity of ATP binding sites, selective and potent inhibition of individual kinases has been achievable for both infectious and non-infectious diseases [6,7]; thus, kinases as a class are considered “druggable.” Furthermore, the *Plasmodium* kinome includes many potentially exploitable differences with respect to the human kinome [8], and kinome-wide essentiality data [9,10] further enable prioritization of possible *Plasmodium* kinase targets.

Based on these considerations and precedents for successful soluble expression [11–14], we selected five *P*. *falciparum* kinases (Table 1) with which to screen cell-active compound collections from GlaxoSmithKline (GSK), St. Jude Children’s Research Hospital, and the Medicines for Malaria Venture (MMV). Two of the kinases, CDPK4 and PK7, are not essential in the erythrocyte stages of the life cycle and thus are unlikely to be any compound’s primary target in these stages. However, an ideal malaria drug is active against multiple life-cycle stages—for example, inhibiting CDPK4 or PK7 in the sexual (gametocyte-to-oocyst) stages and acting at some other target(s) in the erythrocyte stages. Such dual- activity compounds could further the malaria eradication agenda [15] by both treating clinical malaria and blocking transmission.

**Table 1:** *P*. *falciparum* protein kinases selected for biochemical high-throughput screening.

Target name (abbreviation)	PlasmoDB ID (old ID)	Key evidence for importance
Calcium-Dependent Protein Kinase 1 (CDPK1)	PF3D7_0217500 (PFB0815w)	Essentiality in erythrocyte stages of the life cycle has been validated genetically and chemically [16,9].
Calcium-Dependent Protein Kinase 4 (CDPK4)	PF3D7_0717500 (PF07_0072)	CDPK4 is essential in gametocytes, though not in erythrocyte stages [17,9,10].
Mitogen-Associated Protein Kinase 2 (MAPK2)	PF3D7_1113900 (PF11_0147)	MAPK2 is essential in erythrocyte stages (in *P*. *falciparum*, though not *P*. *berghei*) and in male gametogenesis [18–20].
Protein Kinase 6 (PK6)	PF3D7_1337100 (MAL13P1.185)	PK6 is essential in erythrocyte stages [9,10].
Protein Kinase 7 (PK7)	PF3D7_0213400 (PFB0605w)	PK7 knockouts fail to complete oocyst development [21]. They also grow slowly in erythrocyte stages, though PK7 is not essential in these stages [9,10].

## Methods

### Compounds

Collections of anti-*Plasmodium* compounds described in previous reports [3,4,22] were generously provided by GlaxoSmithKline (the 13,000-compound Tres Cantos Antimalarial Set, or TCAMS), St. Jude Children’s Research Hospital (260 compounds), and the Medicines for Malaria Venture (the 400-compound Malaria Box). Compounds for primary screens were provided as 1 mM stocks in dimethyl sulfoxide (DMSO), with 25 nL/well lyophilized in assay-ready plates (GSK/TCAMS); as 50 μM stocks in assay buffer (St. Jude); and as 10 mM stocks in DMSO (MMV). Compounds for dose-response studies were provided as 1000X stocks in DMSO, with 25 nL/well lyophilized in assay-ready plates (GSK/TCAMS).

### Possible kinase substrates and control inhibitors

Bovine proteins β-casein, histone H2A, histone III-S, and myelin basic protein (MBP), as well as Poly(Glu,Tyr), staurosporine, and GW8510 were purchased from Sigma-Aldrich (St. Louis, MO, USA). MEK-1 peptide substrate ADPDHDHTGFLTEYVATRWRR as well as kinase inhibitors 1NA-PP1 and adenosine 5′-(β,γ-imido)triphosphate (AMP-PNP) were purchased from Santa Cruz Biotech (Dallas, TX, USA). Peptide RRRKKSPRKRA was from Genscript (Piscataway, NJ, USA); Syntide-2 (PLARTLSVAGLPGKK) was from American Peptide Company (Sunnyvale, CA, USA). Inhibitors BRB-796, hymenialdisine, and olomoucine were from Calbiochem (now part of EMD Millipore).

### Expression and purification of proteins

Genes for each kinase listed in Table 1 were cloned, expressed, and purified essentially as previously described [12,23,14]. An expression plasmid for PK6 was provided by Christian Doerig. Full-length proteins with N-terminal 6-Histidine tags were purified via nickel affinity column chromatography.

### General biochemical screening assay format

Biochemical assays were performed in white 384-well plates (Nunc). Liquid transfers were done manually with 8-channel pipets. Compounds were initially tested in a single-point screen (final concentration 1 μM). Compounds exhibiting >50% inhibition at 1 μM were then tested at concentrations of 10, 3.3, 1.1, 0.37, 0.12, 0.041, 0.014, 0.0046, 0.0015, 0.0005, and 0.00017 μM to determine the concentration at which 50% inhibition occurs (IC_50_). (In the PK6 and PK7 screens, Z’ factors were suboptimal, so apparent hits were retested at 1 μM before proceeding with dose-response tests.) All incubations were at room temperature (~20°C). The catalytic activity of each kinase was considered proportional to ATP consumed, as determined from measurements of residual [ATP] with the luciferase-based reagent Kinase-Glo (Promega) following incubation. Luminescence (proportional to residual [ATP]) was measured on the plate readers FLx800 (BioTek Instruments, Winooski, VT, USA) and MicroBeta^2^ (PerkinElmer, Waltham, MA, USA). Incubation times were chosen to ensure near-complete ATP depletion in the absence of inhibition, thus maximizing the difference in signal between inhibited and uninhibited wells.

### CDPK1 and CDPK4 screens

As described previously [14], final concentrations were 10 μM ATP, 0.1% bovine serum albumen (BSA) (w/v), 10 mM MgCl_2_, 2 mM CaCl_2_, and 6.6 nM or 208 nM of CDPK1 and CDPK4, respectively, in a buffer of 20 mM HEPES (pH 7.5). 40 μM Syntide-2 was used as a substrate; BKI-1 [14] was used as a control inhibitor, while reaction mixtures lacking Syntide-2 were included as an additional negative control. Incubation time was 90 minutes. Reactions were terminated by adding excess EGTA (5 mM), which sequesters Ca^2+^ and halts calcium-dependent kinase activity.

### MAPK2 screen

Final concentrations were 1 μM ATP, 0.5 mM DTT, 1 mM MgCl_2_, 0.5 mg/mL BSA, and 10 μg/ml MAP2 in a buffer of 50 mM HEPES (pH 7.0). 0.5 mg/mL histone III-S served as the substrate [11]; 100 μM AMP-PNP, an ATP analog, was used as a control inhibitor. Incubation time was 4 hours.

### PK6 screen

Final concentrations were 1.5 μM ATP, 5 mM MnCl_2_, and 15 μg/ml PK6 in a buffer of 100 mM Tris-HCl (pH 7.5). 50 μg/mL MBP was provided as the substrate [12]; 10 μM staurosporine was used as a control inhibitor. Incubation time was 3 hours and 40 minutes.

### PK7 screen

Final concentrations were 1 μM ATP, 2 mM DTT, 20 mM MgCl_2_, 2 mM MnCl_2_, 0.01% BSA, and 6 μg/ml PK7 in a buffer of 20 mM Tris-HCl (pH 7.5). The enzyme itself was the only substrate present (since autophosphorylation occurs [13]); 100 μM 1NA-PP1 [24] was used as a control inhibitor. Incubation time was 3 hours.

### Determination of PK7’s K_m_

To estimate PK7’s K_m_ for ATP, we coupled its ADP production to ADP use by pyruvate kinase (PK) and subsequent oxidation of NADH by lactate dehydrogenase (LDH). The rate at which absorbance at 340 nm decreased was considered indicative of PK7 activity. Aside from the reagents listed above, these assays also included 0.15 NADH, 0.244 mM phosphoenolpyruvate (PEP), 5 U/mL PK, 7.5 U/mL LDH, and ATP concentrations ranging from 3 mM to 1.37 μM.

### ^32^P labeling assays

To confirm that inhibition of ATP consumption reflected inhibition of substrate phosphorylation, selected PK6 inhibitors were studied in ^32^P labeling assays conducted in parallel to the luminescence-based assays. Concentrations were as noted above, with compounds present at 1 μM, except that 8 μCi/mL γ-^32^P ATP was used in place of the usual 1.5 μM unlabeled ATP. Incubation time was 1 hour. 4.6 μL from each well was spotted onto a negatively charged phosphocellulose membrane, attracting the positively charged MBP substrate. The membrane was washed three times for 10 minutes with 0.5% phosphoric acid, dried, and exposed to a phosphoscreen that was scanned with a Typhoon FLA 9000 (GE). Radioactivity on the membrane was considered proportional to phosphorylation of MBP with ^32^P ATP.

### Chemoproteomics

Kinobead competition assays were employed to determine the selectivity of selected hit compounds against the human and *P*. *falciparum* kinomes. Kinobeads were prepared as described [25,26]. The chemoproteomic inhibition binding experiments were performed as previously described [27]. Briefly, Kinobeads were washed and equilibrated in lysis buffer (50 mM Tris-Cl, pH 7.4, 0.4% Igepal-CA630, 1.5 mM MgCl_2_, 5% Glycerol, 150 mM NaCl, 25 mM NaF, 1 mM Na_3_VO_4_, 1 mM DTT, and 1 complete EDTA-free protease inhibitor tablet (Roche) per 25 ml). They were incubated at 4°C for 1 hour with 1 ml (5 mg) K562 extract, which was pre-incubated with compound or DMSO (vehicle control). Beads were transferred to disposable columns (MoBiTec), washed extensively with lysis buffer and eluted with SDS sample buffer. Proteins were alkylated, separated on 4–12% Bis-Tris NuPAGE (Life Technologies), stained with colloidal Coomassie, and quantified by isobaric tagging and LC-MS/MS. Digestion, labeling with TMT isobaric mass tags, peptide fractionation, and mass spectrometric analyses were performed essentially as described [27]. Criteria for protein quantification were: a minimum of 2 sequence assignments matching to unique peptides was required (FDR for quantified proteins <<0.1%), Mascot ion score > 15, signal to background ratio of the precursor ion > 4, signal to interference > 0.5 [28]. Reporter ion intensities were multiplied by ion accumulation time, yielding an area value proportional to the number of reporter ions present in the mass analyzer. Peptide fold changes were corrected for isotope purity as described and adjusted for interference caused by co-eluting nearly isobaric peaks as estimated by the signal-to-interference measure [29]. Protein quantification was achieved using a sum-based bootstrap algorithm [30]. Apparent dissociation constants were determined by taking into account the protein depletion by the beads as described [27].

### Data analysis, statistics, and archiving

K_m_ was calculated from Lineweaver-Burk plots. IC_50_ values were generally calculated from dose-response curves of three independent experiments with Prism 3 software (GraphPad). Z’ factors were calculated as described by Zhang et al. [31]. The parts of Additional File 1 representing our newly reported data have been [will be] deposited in ChEMBL.

## Results

Optimization of CDPK1, CDPK4, and PK6 assays was conducted previously ([14] and C. Bodenreider, unpublished data); here we note highlights of MAPK2 and PK7 assay development.

### MAPK2 characterization and assay development

Our stock of recombinant MAPK2 showed little activity at 37°C in comparison with room temperature (~20°C). 1% glycerol enhanced MAPK2 activity but was left out of the screening assay for simplicity. Since the *P*. *falciparum* MAPK2 may share some features with cyclin-dependent kinases (CDKs), various CDK inhibitors (hymenialdisine, GW8510, olomoucine) as well as the MAP kinase inhibitor BIRB796 were tested for possible use as a control inhibitor. None gave consistent inhibition at concentrations of 10–30 μM, so the ATP analog AMP-PNP was used instead.

### PK7 characterization and assay development

Determining a kinase’s affinity for ATP helps one assess the likelihood of finding compounds that can compete directly with ATP in its active site. The K_m_ of PK7 for ATP has not been reported previously; our assays yielded a value of 46 ± 3 μM. We also looked for protein or peptide substrates that would enhance PK7’s baseline rate of ATP consumption (the baseline rate presumably being due to PK7 autophosphorylation). MBP, β-casein, and histone H2A have been reported to be phosphorylated by PK7 in vitro [13] but did not noticeably augment PK7’s intrinsic ATPase activity. The same was true of peptide RRRKKSPRKRA, which conforms to a previously reported consensus sequence (though the consensus sequence was not itself tested as a substrate) [24]; the peptide ADPDHDHTGFLTEYVATRWRR, a substrate of MEKs (a.k.a. MAP kinase kinases), to which PK7 is related; and the generic tyrosine kinase substrate Poly(Glu,Tyr), which was tried for completeness, despite uncertainties about tyrosine phosphorylation in *Plasmodium* [32]. In the absence of an ATPase-enhancing substrate, we screened compounds for their ability to inhibit PK7’s intrinsic ATPase activity, presumably reflecting autophosphorylation.

### High-throughput screening

The ~14,000 anti-*Plasmodium* compounds noted above were tested for possible inhibition of the five *P*. *falciparum* protein kinases noted above. Table 2 provides an overview of the screens. Z’ factors (based on 16 positive and 8–16 negative control wells per plate) were generally good, especially considering that all pipetting was done manually. While no hits were obtained for MAPK2 and only 2 hits were found for PK7, numerous hits were identified for CDPK1, CDPK4, and PK6 (Table 2).

**Table 2:** Summary of biochemical screens of *P*. *falciparum* protein kinases.

Target	median Z’	# of initial hits (hit rate)	# of confirmed sub-μM hits
CDPK1	0.71	220 (1.6%)	181
CDPK4	0.84	77 (0.6%)	56
MAPK2	0.66	0 (0%)	0
PK6	0.34	83 (0.6%)	65
PK7	0.48	2 (0.01%)	2

**Fig 1:**
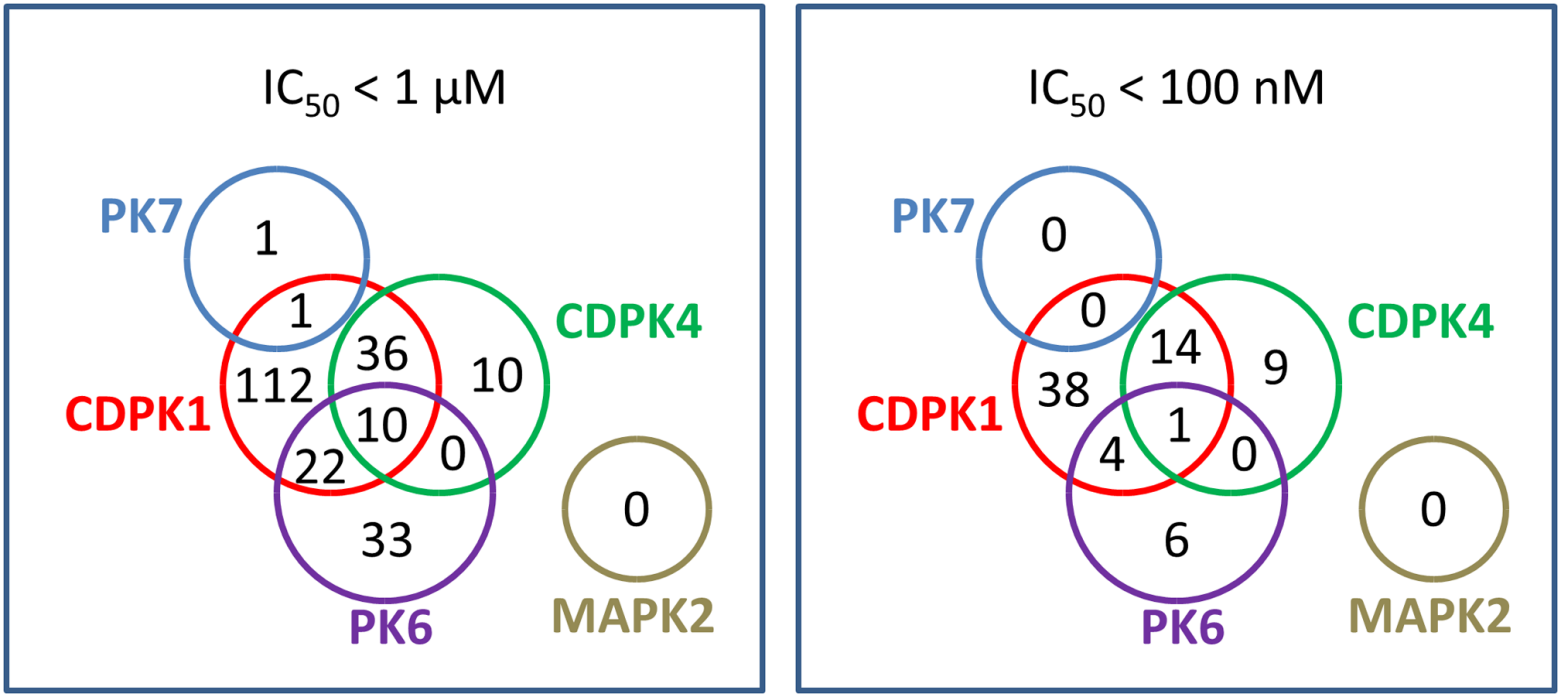
Venn diagrams showing overlapping and non-overlapping targets of hit compounds. 225 compounds had IC_50_’s below 1 μM against at least one kinase (left); a subset of 72 compounds had IC_50_’s below 100 nM against at least one kinase (right).

As might have been expected from the similarity of the ATP binding sites in CDPK1 and CDPK4 [14], there was considerable overlap among the compounds that inhibited CDPK1 and those that inhibited CDPK4 (Fig 1). More surprisingly, many inhibitors acted both against CDPK1 and PK6. A complete listing of all sub-micromolar hits is given in S1 File.

### Independent confirmation of inhibition

**Fig 2:**
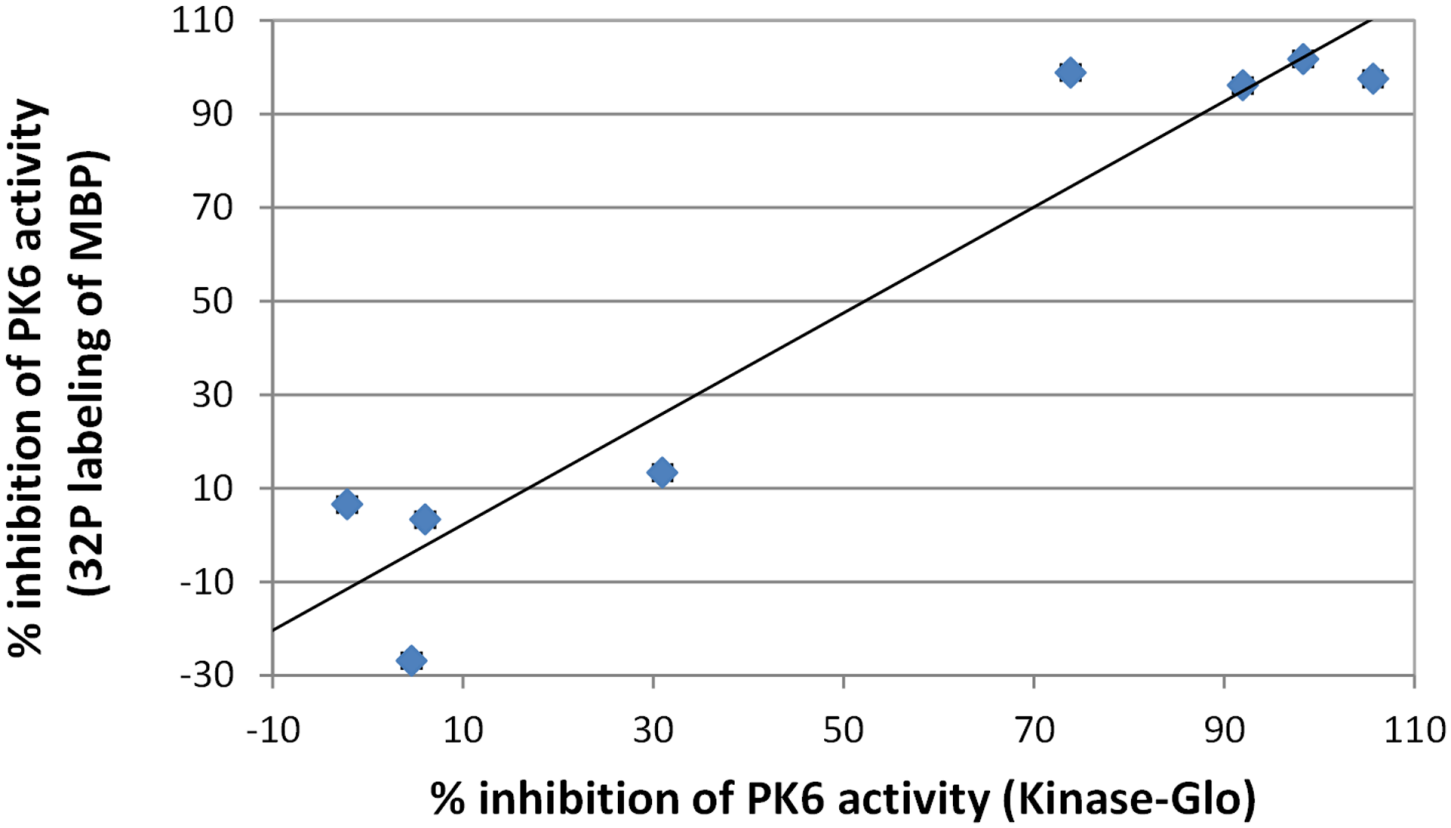
Correlation of ATP depletion (measured with Kinase-Glo) with MBP phosphorylation (measured with ^32^P-ATP). Eight compounds were studied for possible inhibition of PK6. Compounds that impaired ATP use also impaired MBP phosphorylation. Each data point represents an average of two independent experiments conducted on separate days.

Since our screening assays measured ATP consumption rather than phosphorylation per se, we sought to confirm that inhibition of ATP use would reflect inhibition of phosphorylation. Low-throughput tests of compounds that did and did not inhibit PK6 revealed a strong correlation (R^2^ = 0.91) between inhibition of ATP consumption and inhibition of MBP phosphorylation as measured via ^32^P labeling (Fig 2).

### Analysis of scaffolds

**Fig 3:**
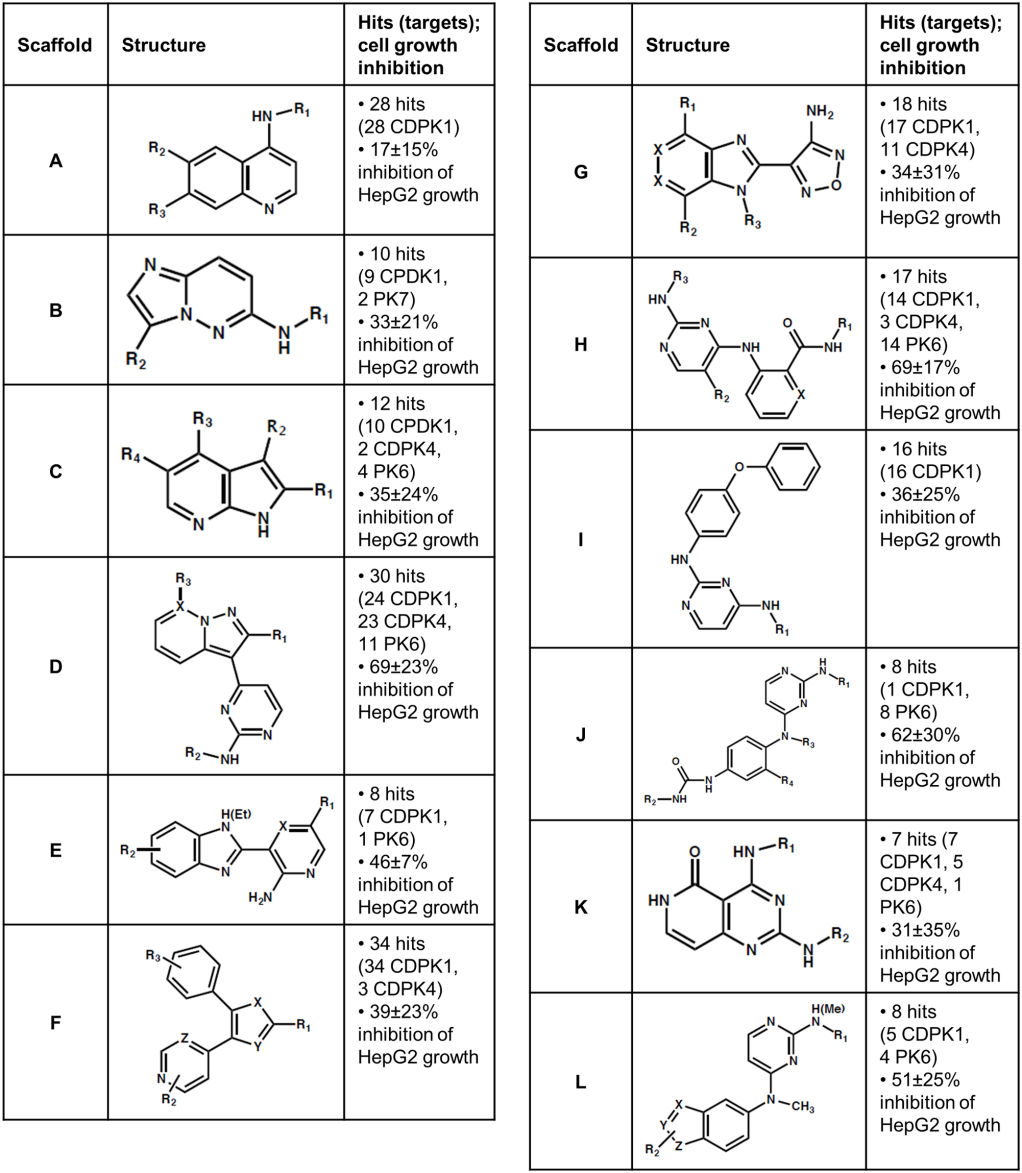
Clustering of *P*. *falciparum* protein kinase hits into chemical scaffolds. Inhibition of HepG2 cell growth at compound concentrations of 10 μM were previously reported by Gamo et al. [3]. For some scaffolds, target counts exceed the number of hits because some compounds hit more than one target.

Structurally related hits were clustered manually into scaffolds. 196 hits were grouped into scaffolds A through L (Fig 3), while the other 29 hits were either singletons or members of very small clusters (<6 members). Our clusters were broadly consistent with those generated previously [3], though our tendency was toward fewer distinct scaffolds with more members. For instance, scaffold A includes members of 7 clusters previously generated with molecular frameworks and members of 9 clusters previously generated with Daylight fingerprinting (Additional File 1).

Many of the compounds covered by Fig 3 were previously recognized as likely *P*. *falciparum* kinase inhibitors based on their effects on human kinases [3]. For example, scaffold D inhibits Epidermal Growth Factor Receptor (EGFR), scaffold G inhibits Akt (a.k.a. Protein Kinase B), and scaffold K inhibits Pyruvate Dehydrogenase Kinase (PDK); it is likely that these scaffolds also target one or more *P*. *falciparum* kinases structurally similar to the human kinases. On the other hand, none of scaffold I’s 16 compounds and only 1 of scaffold B’s 10 compounds were previously annotated as probable kinase inhibitors based on historical screening data [3]. The finding that these scaffolds inhibit at least one *P*. *falciparum* kinase (primarily CDPK1, so far) thus constitutes additional progress toward identifying their specific target(s).

### Integration of new data with previous data

**Fig 4:**
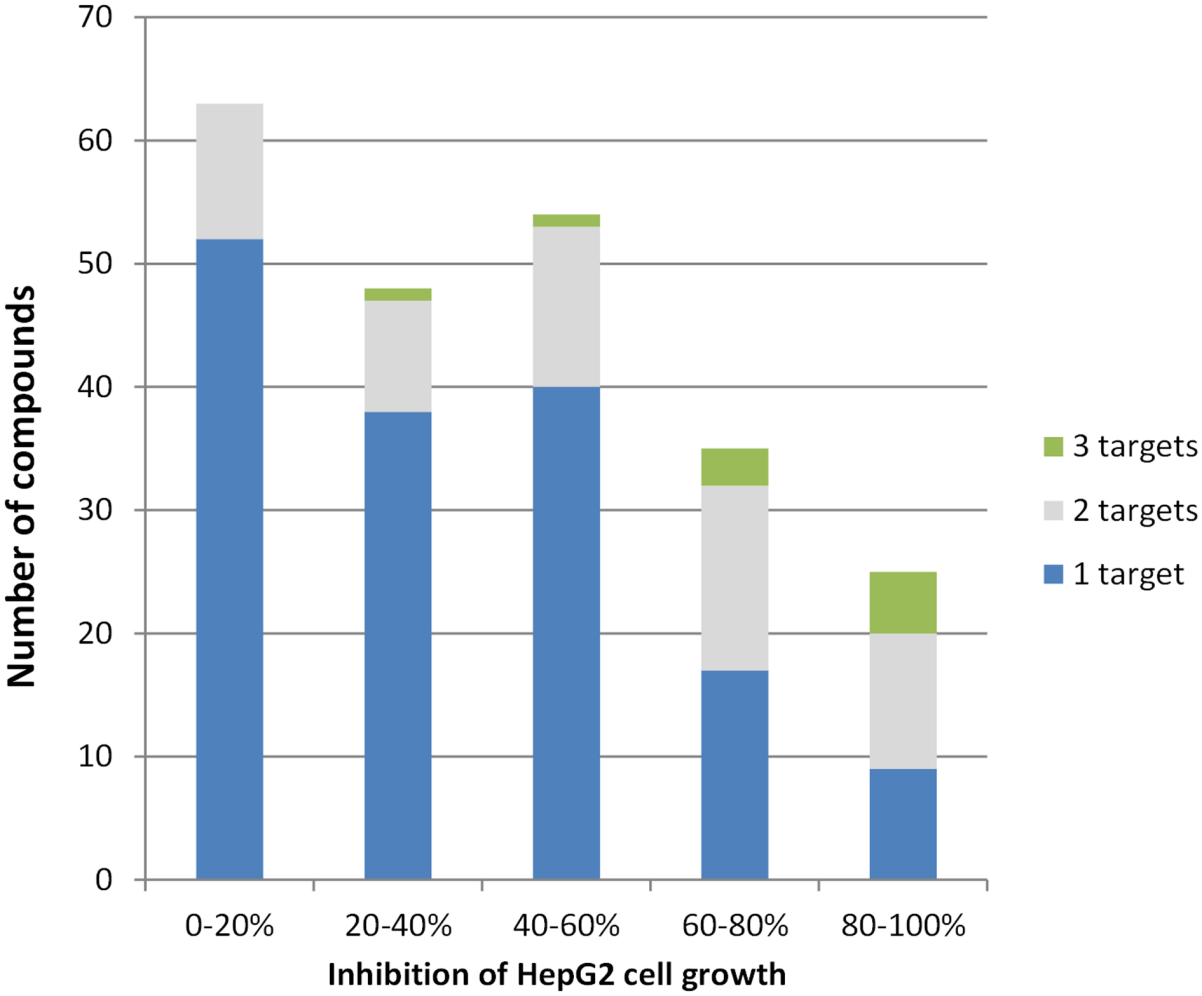
Human cytotoxicity of inhibitors of 1, 2, or 3 of the *P*. *falciparum* kinases studied. Inhibition of HepG2 cell growth at compound concentrations of 10 μM were previously reported by Gamo et al. [3].

The results reported here add to what is already known about these publicly disclosed antimalarial compounds, permitting further insights. For example, regarding the possibility of a compound that inhibits multiple *Plasmodium* kinases and thus acts at multiple life-cycle stages, we can ask whether multi-kinase inhibitors are inevitably cytotoxic. Fig 4 shows that inhibitors of multiple kinases do tend to be more cytotoxic to human HepG2 cells; however, many of the dual-activity compounds have low to moderate cytotoxicity at a concentration of 10 μM.

**Fig 5:**
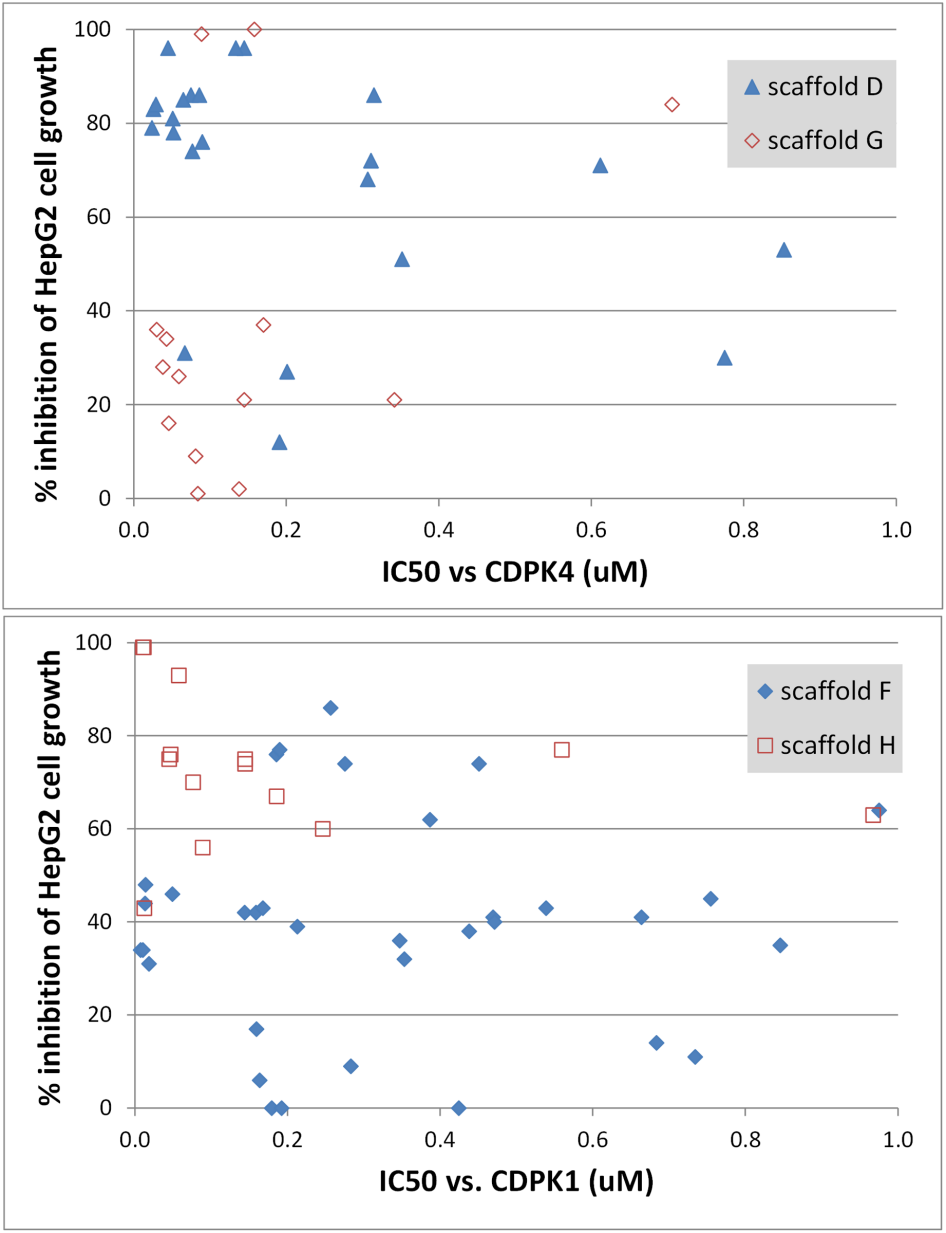
A comparison of different CDPK inhibitors’ cytotoxicity to human cells. Inhibition of HepG2 cell growth at compound concentrations of 10 μM is shown for CDPK4 inhibitors in scaffolds D and G (top) and for CDPK1 inhibitors in scaffolds F and H (bottom).

In terms of cytotoxicity, some scaffolds look more appealing than others. A comparison of scaffolds D and G revealed that, while compounds in both scaffolds cover a wide range of potencies against CDPK4, most scaffold-D compounds are fairly cytotoxic to human HepG2 cells, whereas most scaffold-G compounds are more benign (Fig 5). A similar trend can be seen with scaffold-F and scaffold-H inhibitors of CDPK1; members of scaffold F generally appear less cytotoxic to HepG2 cells (Fig 5). These data may be useful in prioritizing scaffolds during drug development.

Correlations between compounds’ IC_50_s against a given molecular target and EC_50_s against *P*. *falciparum* cell growth, if found, would suggest that the target may indeed represent the compounds’ primary mechanism of action. However, IC_50_s against CDPK1, CDPK4 and PK6 did not strongly correlate with previously reported EC_50_s against *P*. *falciparum* 3D7 cells, either overall (S1 Fig; R^2^≤0.1 for each enzyme) or within individual scaffolds (data not shown). These poor correlations could be due to one or more of several factors, such as (A) the limited ranges of IC_50_s and EC_50_s studied; (B) polypharmacology, i.e., compounds’ actions at additional (unknown) targets, perhaps (unlike CDPK4 and PK7) expressed strongly in the erythrocyte life-cycle stages tested in the EC_50_ assays; and (C) compounds’ varied bioavailability in the EC_50_ assays, e.g., due to variations in membrane permeance or binding to components of blood used in the EC_50_ assays.

### Assessment of compound promiscuity

The possibility that an inhibitor might target multiple *Plasmodium* kinases but leave the human kinome untouched, however appealing, is difficult to evaluate with biochemical assays alone. Moreover, while strong inhibition of HepG2 cell growth *might* indicate that a compound inhibits human kinases as well, such growth inhibition might also involve mechanisms unrelated to kinases. To obtain direct profiles of selected compounds’ activity against the *P*. *falciparum* and human kinomes, we tested these compounds in kinobead competition assays.

**Fig 6:**
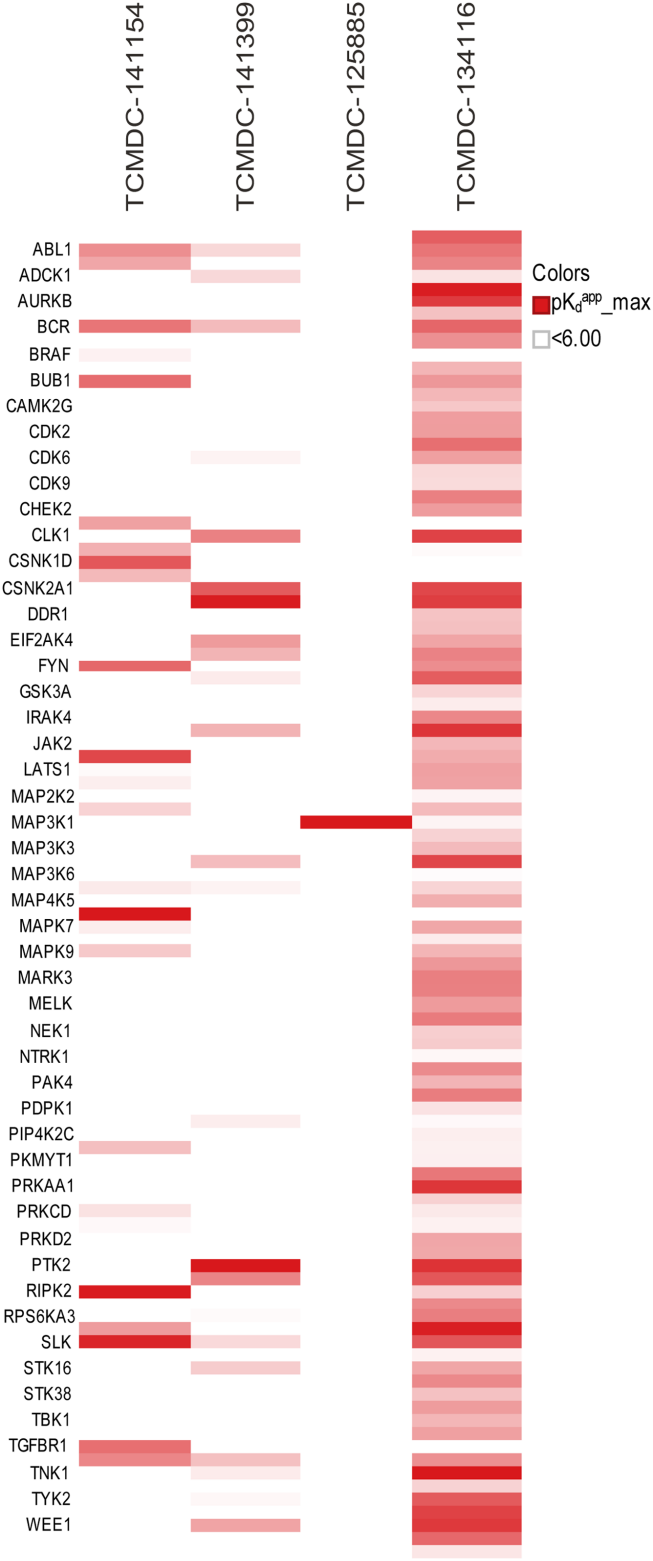
Assessment of compound promiscuity with human kinases. Kinobeads were incubated with K562 cell extract either in the presence of vehicle (DMSO) or TCAMS compound, respectively (20 μM-0.03 μM). Protein kinases captured by the beads (140–150 kinases per experiment) were quantified following tryptic digestion, isobaric peptide tagging, and LC-MS/MS analysis. Kinases were identified as potential targets by virtue of their reduced capture in the presence of excess TCAMS compounds. Apparent dissociation constants (K_d_’s) were calculated from the extent to which capture of each kinase was reduced at each compound concentration. K_d_ values from duplicate experiments generally agreed with each other quite well (S2 Fig). Colored bands indicate kinase-ligand complexes with apparent pK_d_’s of ≥6, with darker shades denoting higher pK_d_’s. Kinases that did not have an apparent pK_d_ of ≥6 for any of the compounds are not represented; only names of every other targeted kinase are shown due to space limitations. These results are summarized numerically in Table 3.

The kinobead results are illustrated in Fig 6 and summarized numerically in Table 3. Interestingly, although the four tested compounds had similar effects on HepG2 cell growth, inhibiting it by 33–48% at 10 μM, their effects on human kinases varied greatly. At the extremes, TCMDC-12885 had a “clean” profile, while TCMDC-134116 bound to two thirds of the human kinases tested with high affinity (pK_d_ ≥ 6).

**Table 3:** Summary of kinobead competition assays (results reflect two independent experiments).

Compound	Inhibition of HepG2 cell growth at 10 μM [3]	Human kinases quantified on kinobeads (N = 2)	Human kinases with apparent pK_d_ ≥ 4.7 (N = 2)	Human kinases with apparent pK_d_ ≥ 6 (N = 2)
TCMDC-125885	33%	153	5	1
TCMDC-134116	43%	136	126	96
TCMDC-141154	48%	146	47	26
TCMDC-141399	47%	147	63	24

## Discussion

We attained mixed results in our effort to link anti-*Plasmodium* compounds with possible *P*. *falciparum* protein kinase targets. At one extreme, none of the 14,000 compounds gave significant inhibition of MAPK2 or PK7, with the exception of two members of an imidazopyridazine scaffold previously reported to inhibit PK7 and many other kinases [33]. These findings could mean that (A) our stocks of MAPK2 and/or PK7 were not well-folded; (B) sampling of chemical space was inadequate for finding inhibitors; (C) these enzymes are not very druggable; and/or (D) these enzymes adopt different, more druggable conformations in a cellular context with interacting with other proteins. We believe that explanation (A) is unlikely to be true, in part because thermal melt assays of MAPK2 and PK7 (data not shown) revealed characteristics of stable proteins (low fluorescence at baseline, high melting temperature, large change in fluorescence during melting) [34]. Explanation (B) also seems somewhat unlikely, at least for MAPK2, because a thermal melt screen of this enzyme against 2,000 diverse kinase inhibitors gave no hit compounds that raised the melting temperature by >2°C (data not shown), whereas an unstable yet druggable protein should have been hit in this screen. Thus, the simplest explanation of our data may be that MAPK2 and PK7 have low druggability in vitro (D) or perhaps in general (C).

On the other hand, our data include the most numerous, diverse, and potent PK6 inhibitors reported to date. Previous studies of PK6 [12,35] have not included extensive searches for chemical inhibitors. Our study thus complements previous reports in revealing many potential “tool compounds” for further study of PK6.

CDPK1 and CDPK4 have been the focus of much recent research, and potent inhibitors of each are already known [36,37,14,38,39]. Specificity of inhibition has been achieved with compounds that are accommodated by these enzymes’ unusually small “gatekeeper residues” in the ATP binding pocket (threonine for CDPK1, serine for CDPK4). The present study provides further biochemical characterization of the CDPK1 and CDPK4 ATP binding sites. Given the similarity of these binding sites in CDPK1 and CDPK4 [14], it surprised us that only 25% of the CDPK1 hits (46 of 181) had sub-micromolar IC_50_s against CDPK4. Since the “gatekeeper residue” is slightly larger in CDPK1 (threonine) than in CDPK4 (serine), it was also surprising that CDPK1 had many more hits than CDPK4 (181 vs. 56). It seems that, in the vicinity of the CDPK1 and CDPK4 gatekeeper residues, compound binding efficacy may be determined at least as much by the strength of interactions with amino acids as by steric limitations of the binding pocket [40].

The data presented here complement previous efforts to link hits from cell-based *P*. *falciparum* screens to specific molecular targets. Of the 76 compounds associated with 16 specific protein targets in previous reports [2,4,41,42], only one compound is a member of any scaffold shown in Fig 3. Scaffold A fits the structure of compound “CK-8” (also called SJ000111331 and GNF-Pf-4995), a modest inhibitor of the *P*. *falciparum* choline kinase (IC_50_ = 12 μM) [41]. Our connection of scaffolds A through L to specific protein kinases establishes novel hypotheses about these scaffolds’ exact sites of action.

As with previous biochemical and biophysical screens of anti-*Plasmodium* compounds [4,41,42], our results do not prove that the kinases studied here are the only or primary targets through which the associated compounds kill *P*. *falciparum* cells. The hypothesis that a specific kinase is a compound’s primary target could be tested by evolving resistance to the compound and determining whether the resistance-enabling mutants occur in the gene of the hypothesized target [43,44].

Finally, our data offer some indication as to the likelihood of developing a multi-kinase-targeting compound that is efficacious against malaria but safe in humans. Most individual compounds that inhibited three kinases were highly cytotoxic to human liver cells, but some dual-acting compounds, especially in scaffold G, were not (Figs 4 and 5). Ease of achieving joint inhibition of CDPK1 and CDPK4 was mixed, as 82% of CDPK4 hits also inhibited CDPK1 but only 25% of CDPK1 hits inhibited CDPK4 (Fig 1). While many of the multi-acting compounds were members of scaffold D, which generally looked cytotoxic (Fig 5), EGFR inhibitors based on this scaffold have been patented as possible lung cancer drugs, so clinically safe compounds based on scaffolds like this one may be possible. On the whole, our data provide a basis for both optimism and cautiousness regarding the concept of a multi-kinase-targeting malaria drug.

## Supporting Information

S1 Fig Correlations between compounds’ pEC_50_ for inhibition of growth of *P*. *falciparum* 3D7 and their pIC50 against CDPK1 (top), CDPK4 (middle), and PK6 (bottom). (PNG)

S2 Fig Correlations between independent determinations of apparent K_d_ of protein-ligand complexes. X and Y axes are both labeled with the negative logarithm of K_d_ values (pK_d_).
(PNG)

S1 File Complete list of inhibitors of CDPK1, CDPK4, PK6, and PK7. This Microsoft Excel file includes IC_50_s, scaffold information, and previously collected data downloaded from ChEMBL (https://www.ebi.ac.uk/chembl/).
(XLS)

S2 File Kinobeads profiling of TCAMS compounds in K562 cell extracts. This Microsoft Excel file includes a table of the experimental setup and apparent dissociation constants determined by taking into account protein depletion by the beads.
(XLSX)

## Abbreviations

AMP-PNP: adenosine 5′-(β,γ-imido)triphosphate
BSA: bovine serum albumen
CDPK1: calcium-dependent protein kinase 1
CDPK4: calcium-dependent protein kinase 4
EGFR: epidermal growth factor receptor
IC_50_: 50% inhibitory concentration
LDH: lactate dehydrogenase
MAPK2: mitogen-associated protein kinase 2
MBP: myelin basic protein
MMV: Medicines for Malaria Venture
*P. falciparum*: *Plasmodium falciparum*
PDK: pyruvate dehydrogenase kinase
PEP: phosphoenolpyruvate
PK: pyruvate kinase
PK6: protein kinase 6
PK7: protein kinase 7
SGC: Structural Genomics Consortium
