# Supplementary figures and images for: Biochemical Screening of Five Protein Kinases from Plasmodium falciparum against 14,000 Cell-Active Compounds
Source: PLoS One. 2016 Mar 2;11(3):e0149996. doi: 10.1371/journal.pone.0149996 (PMC4774911; doi:10.1371/journal.pone.0149996)

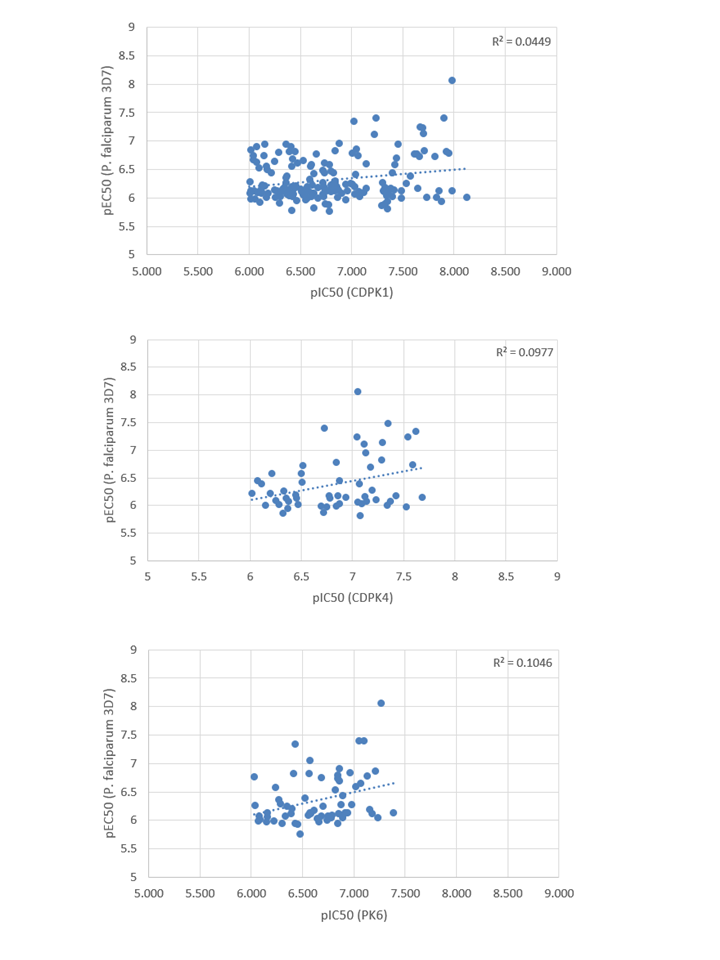

Supplement: S1 Fig — (PNG) [file pone.0149996.s001.png]

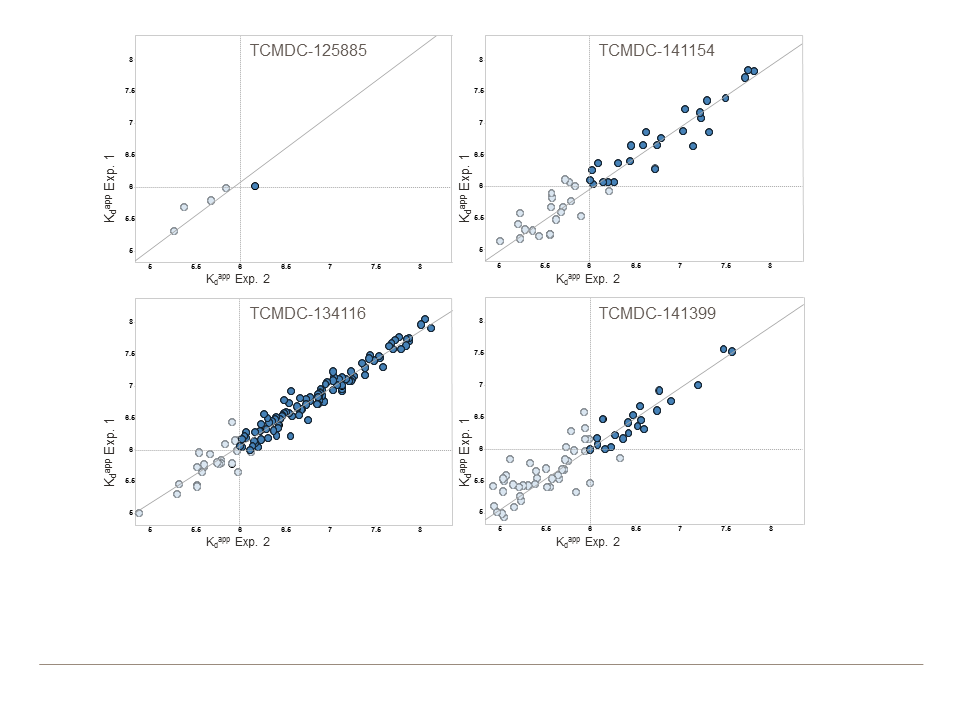

Supplement: S2 Fig — X and Y axes are both labeled with the negative logarithm of Kd values (pKd). (PNG) [file pone.0149996.s002.png]
